# Somatic symptom disorder in gastroesophageal reflux disease: a key factor contributing to severe symptoms and impaired quality of life

**DOI:** 10.3389/fmed.2026.1762738

**Published:** 2026-02-18

**Authors:** Ya Jiang, Li Gao, Weichen Liu, Yurong Tang

**Affiliations:** Department of Gastroenterology, First Affiliated Hospital with Nanjing Medical University, Nanjing, Jiangsu, China

**Keywords:** gastroesophageal reflux disease, psychological distress, quality of life, screening, somatic symptom disorder

## Abstract

**Background:**

Somatic symptom disorder (SSD) is a common yet frequently overlooked comorbidity in patients with gastroesophageal reflux disease (GERD), compromising quality of life (QOL) and increasing healthcare utilization.

**Aims:**

This study aimed to evaluate its impacts of SSD on symptom perception and QOL and to determine the risk factors for SSD in GERD patients.

**Methods:**

A cross-sectional study was conducted in a tertiary gastroenterology department. A total of 209 GERD patients completed validated questionnaires assessing reflux symptoms, somatic symptoms, anxiety, depression, sleep quality, and QOL. SSD was diagnosed based on DSM-5 criteria. All patients underwent upper endoscopy and a subset completed esophageal manometry and 24-h pH-impedance monitoring investigations.

**Results:**

The proportion of SSD was 30.14% (63/209). Despite having milder objective reflux burden on 24-h pH-impedance monitoring and esophageal manometry, patients with SSD reported higher reflux symptom scores [GerdQ: 9(5) vs. 8(4), *p* = 0.032; RSI: 12(9) vs. 4(6), *p* < 0.001], more severe anxiety and depression, worse sleep quality (all *p* < 0.001), and lower QOL [GIQLI: 94(37) vs. 121(27.5), *p* < 0.001] than non-SSD patients. Female gender, laryngopharyngeal reflux symptoms, anxiety, and depression were independent risk factors for SSD. SSD partially mediated the negative impacts of reflux symptoms and poor sleep on QOL (23.52% for GerdQ, 26.30% for RSI and 14.05% for PSQI). The PHQ-15 demonstrated high accuracy (AUC = 0.851) in identifying comorbid anxiety/depression with sleep disturbances at a cutoff score of 12.5.

**Conclusion:**

SSD aggravates GERD symptoms and mediates impaired QOL by reflux and sleep disturbance. Screening SSD with PHQ-15 is recommended, particularly in females and those with laryngopharyngeal reflux symptoms.

## Introduction

Gastroesophageal reflux disease (GERD) is a common condition in gastroenterology clinics of general hospitals, affecting 1.9–7.0% of the Chinese population with a rising prevalence in recent years (1). While its typical symptoms are heartburn and acid regurgitation, a significant clinical challenge lies in the high frequency of psychosomatic comorbidities, such as anxiety, depression, and somatic symptoms (2). A bidirectional Mendelian randomization has demonstrated a bidirectional interplay between physiological and psychological manifestations in GERD (3). The impact of mental disorders on GERD may stem from the gut–brain axis with amplification of the autonomic nervous system, resulting in elevated neuronal hypersensitivity (4, 5). The psychosomatic issues are progressively acknowledged to cause persistent GERD symptoms and reduce treatment efficacy.

In this context, somatic symptom disorder (SSD) is of particular significance. SSD is a condition marked by persistent and distressing physical symptoms that lack a medical explanation (6). SSD frequently remains underdiagnosed by non-psychiatric physicians who are primarily focused on organic disease (6). Notably, approximately 20% of GERD patients exhibit somatoform tendencies, which substantially impairs quality of life (QOL) and leads to increased healthcare utilization (7, 8). SSD lies in dysregulation across cognitive, affective, and behavioral dimensions. Patients exhibit a preoccupation with somatic symptoms, accompanied by excessive health-related anxiety, which causes maladaptive behaviors, like recurrent body checking and frequent medical consultations (6, 9). SSD can be comorbid with anxiety or depression (9). The Patient Health Questionnaire-15 (PHQ-15) (10, 11) is a widely used self-report questionnaire evaluating the severity of somatic symptoms. It helps to quickly recognize patients whose physical symptoms may have a strong psychological component.

Visceral hypersensitivity and altered central pain processing involved in SSD may enhance symptom perception in GERD (12, 13). Existing studies primarily focused on the influence of anxiety and depression on reflux symptoms, leaving the associations between SSD and subjective symptom burden, objective reflux indicators, and QOL insufficiently clarified. Moreover, there is a need for efficient and accurate instruments to evaluate comorbid psychosomatic distress in GERD. To address these gaps, the primary aim of this study was to investigate the impacts of SSD on clinical symptoms, objective reflux evidence and QOL. The secondary aims were to elucidate the risk factors for SSD and to determine the utility of the PHQ-15 in assessing psychological disturbance in this patient population.

## Methods

### Study population

Consecutive patients (aged 18–75 years) with typical GERD symptoms (heartburn and/or reflux ≥2 days/week for >3 months) at the First Affiliated Hospital of Nanjing Medical University (July 2023–December 2024) were screened. Those satisfying diagnostic criteria of Chinese expert consensus on GERD in 2020 (1) were included. Inclusions comprised: (1) reflux esophagitis (Los Angeles grade B/C/D) based on upper endoscopy; (2) responsive to 2-week PPI test; (3) AET > 4% according to 24 h-MII-pH monitoring. Individuals meeting DSM-5 criteria were considered as SSD (6, 14–16). The diagnostic criteria of DSM-5 SSD were as below: A. One or more somatic symptoms that are distressing or disabling somatic symptoms that disrupt daily life. B. Excessive thoughts, feelings, or behaviors related to the somatic. Symptoms as manifested by one or more of the following: (1) disproportionate and persistent thoughts about the seriousness of one’s symptoms (cognitive dimension); (2) high level of anxiety about health or symptoms (affective dimension); (3) excessive energy or time devoted to these symptoms or health concerns (behavioral dimension). Somatic symptoms should persist for over 6 months.

All potential participants first underwent standard clinical evaluation and upper endoscopy. A total of 119 patients had 24 h-multichannel intraluminal impedance (MII)-pH monitoring and 105 patients underwent esophageal manometry. Exclusions comprised prior foregut surgery, organic lesions on upper endoscopy including eosinophilic esophagitis, taking recent acid-suppressive therapy 7 days before or during the testing or taking anti-depressants. Patients with severe dysphagia, vomiting or endoscopic abnormalities, such as dilated esophagus, contraction rings or food retention, were excluded. Additionally, individuals suspected of esophageal motility disorders underwent esophageal manometry and those diagnosed with achalasia, distal esophageal spasm, Jackhammer esophagus or esophagogastric junction (EGJ) outflow obstruction were subsequently excluded. The study protocol adhered to the Declaration of Helsinki and was approved by the Institutional Ethics Committee (2024-SR-682). Written informed consent was obtained from all the enrolled patients.

### Clinical assessments

#### Questionnaires

We employed validated questionnaires to evaluate reflux symptoms, somatic symptoms, sleep quality, anxiety, depression and QOL. A score of 10 or more on the Generalized Anxiety Disorder 7-item (GAD-7) scale (17, 18) and the 9-item Patient Health Questionnaire (PHQ-9) (18, 19) were used to identify patients with anxiety and depression in outpatients. A score more than 5 on the Pittsburgh Sleep Quality Index (PSQI) was applied to recognize undermined sleep (20). The score of PHQ-15 indicated the severity of SSD with higher scores showing more severe somatic symptoms (10). We used the GerdQ to assess gastroesophageal reflux symptoms (21) and reflux severity index (RSI) to assess laryngopharyngeal reflux symptoms (22). QOL was evaluated by the Gastrointestinal Quality of Life Index (GIQLI) carrying 5 different domains such as gastrointestinal symptoms, emotional factors, physical factors, social factors and influences by the treatment, which is well established in a wide range of patient groups (23). Higher scores represent more worsened QOL.

#### Procedures

The patients had upper endoscopy according to international guidelines before 24-h pH-impedance monitoring and esophageal manometry.

Esophageal manometry (Given Imaging) was employed to locate the lower esophageal sphincter (LES) and exclude major esophageal dysmotility ahead of 24-h pH-impedance monitoring. 36-channel solid-state catheter with pressure sensors was inserted by a trained nurse, in a 30 to 45-degree-recumbent position after overnight fast. The manometric protocol included a 30-s baseline recording and 10 swallows of 5 mL water at 30-s intervals. The studies were analyzed manually by two independent experienced physicians through using the Manoview software (Given Imaging). Esophagogastric junction (EGJ) indicators and peristalsis indicators of esophagus body were collected (24). Esophageal manometry can detect physiological abnormalities associated with GERD such as a low LES pressure, or weak/absent peristalsis (24). Decreased esophageal motility suggested by lower LES pressure and distal contractile integral (DCI), fewer normal contractions and more ineffective contractions may contribute to poor clearance of reflux matter.

24-h pH-impedance monitoring was performed with the ambulatory monitoring system (Given Imaging). This system consists of a portable data logger with impedance-pH amplifiers and a catheter containing one pH channel and six impedance channels. The pH electrodes were calibrated using pH 4.0 and pH 7.0 buffer before monitoring. The catheter was placed through the nostril to distal esophagus with pH electrode 5 cm above the LES located by manometry device. After placing the catheter, patients returned to daily diet and activities. The percentage of acid exposure time, Demeester score and reflux episodes were collected (24).

### Statistical analysis

The data were analyzed using SPSS version 27.0 (IBM, Armonk, NY, USA). For quantitative variables, the Mann–Whitney *U*-test was employed as the data were non-normally distributed. These data were presented as median (inter-quartile range, IQR). Categorical variables were subjected to chi - square tests. Multivariate and logistic regression analyses were conducted to identify the risk factors for SSD. Mediation analysis was employed to investigate whether SSD mediates impaired QOL by the reflux symptoms and psychological distress. Receiver operating characteristic (ROC) curves were used to assess the predictive score of PHQ - 15 for poor sleep (PSQI score >5) and anxiety/depression (GAD-7/PHQ-9 score ≥10). Area Under the Curve (AUC) represents the ability to measure overall differences between two groups by the models. A two - sided *p* < 0.05 was regarded as statistically significant.

## Results

### Demographic characteristics

A total of 209 eligible patients with GERD were enrolled to the final analysis, among whom 63 (30.14%) were diagnosed with comorbid SSD. No significant intergroup difference was observed in age, body mass index (BMI), or disease duration. However, the SSD group contained a significantly higher proportion of females (66.67% vs. 45.21%, *p* = 0.004) (Table 1).

**Table 1:** Demographic characteristics, clinical symptoms and QOL in GERD patients.

Clinical variables	GERD with SSD (*n =* 63)	GERD without SSD (*n =* 146)	Effect size	*p*-value
Demographics				
Age (yr)	45 (22)	46 (22.25)	0.038	0.582
Gender, female (%)	42 (66.67%)	66 (45.21%)	0.197	0.004
BMI (kg/m^2^)	22.77 (5.14)	23.52 (4.91)	0.088	0.204
GERD duration (yr)	2.5 (3)	2 (3.53)	0.063	0.359
Reflux				
GerdQ score	9 (5)	8 (4)	0.149	0.032
RSI score	12 (9)	4 (6)	0.464	<0.001
Psychological distress				
GAD-7 score	13 (7)	4.5 (7)	0.571	<0.001
PHQ-9 score	14 (11)	4 (5)	0.58	<0.001
PHQ-15 score	14 (5)	4 (4)	0.795	<0.001
Sleep				
PSQI score	10 (6)	6 (5)	0.424	<0.001
Sleep quality	2 (2)	1 (0)	0.409	<0.001
Time to fall asleep	2 (2)	1 (1)	0.27	<0.001
Sleep duration	2 (1)	1 (2)	0.263	<0.001
Sleep efficiency	1 (2)	1 (2)	0.039	0.574
Sleep disturbance	2 (1)	1 (0)	0.495	<0.001
Drugs usage	0 (0)	0 (0)	0.091	0.19
Daytime dysfunction	2 (1)	2 (1)	0.508	<0.001
Quality of Life				
GLIQI	94 (37)	121 (27.5)	0.471	<0.001

Data are presented as frequencies (%) and median (inter-quartile range, IQR). Data are analyzed by chi-squared test or Mann–Whitney *U*-test. Effect sizes: r for quantitative data, Φ (phi) for categorical data (gender). There were more females in SSD group. Statistically significant more reflux symptoms were found by GerdQ and RSI in patients with SSD; GAD-7, PHQ-9 showed higher scores in GERD with SSD group; Sleep quality and quality of life were dramatically reduced in SSD patients suggested by PSQI and GLIQI scores. BMI, body mass index; yr, year; GerdQ, gastroesophageal reflux disease questionnaire; RSI, reflux symptom index; GAD-7, Generalized Anxiety Disorder 7-item; PHQ-9, 9-item Patient Health Questionnaire; PSQI, Pittsburgh Sleep Quality Index; GLIQI, Gastrointestinal Quality of Life Index.

### Reflux and esophageal motility profiles of GERD patients with/without SSD

Patients with GERD and SSD reported significantly higher symptom scores on both GerdQ [9(5) vs. 8(4), *p* = 0.032] and RSI [12(9) vs. 4(6), *p* < 0.001] compared to those without SSD (Table 1). GerdQ and RSI scores did not differ significantly between males and females in the overall study population, nor within the SSD or non-SSD groups (all *p* > 0.05). Surprisingly but interestingly, objective indicators derived from 24-h pH-impedance monitoring revealed a milder reflux burden in the SSD group, as indicated by lower upright acid exposure time (AET), total AET and Demeester scores (all *p* < 0.05, Table 2). The motility impairments of esophagus, including reduced LES pressure and DCI, fewer normal peristaltic contractions, and more ineffective contractions, were observed in patients with non-SSD compared to those with SSD (all *p* < 0.05, Table 3). These results indicated that patients with SSD suffered from less objective reflux burden, despite more severe symptoms.

**Table 2:** Reflux evidence from 24 h-MII-pH monitoring in GERD patients.

Indicators	GERD with SSD (*n =* 37)	GERD without SSD (*n =* 82)	Effect size	*p*-value
Upright AET (%)	1.1 (4.45)	2.1 (5.5)	0.22	0.016
Recumbent AET (%)	0 (0.7)	0.3 (1.65)	0.138	0.132
Total AET (%)	0.6 (2.7)	1.9 (4.15)	0.222	0.015
Demeester Score	3 (11)	6.3 (14.7)	0.213	0.02
Reflux episodes (acid)	14 (25)	18 (24)	0.151	0.099
Reflux episodes (weak acid)	10 (10.5)	12 (14.5)	0.039	0.671
Reflux episodes (weak base)	0 (2)	1 (2)	0.103	0.26
Total reflux episodes	27 (27)	35 (35.5)	0.138	0.132

Data are presented as median (interquartile range, IQR). Data are analyzed by Mann–Whitney *U*-test. Effect sizes: r. Less upright AET, total AET and lower Demeester score were found in patients with SSD than those without SSD. AET: acid exposure time.

**Table 3:** The esophagus motility characteristics in GERD patients.

Indicators	GERD with SSD (*n =* 33)	GERD without SSD (*n =* 72)	Effect size	*p*-value
LES resting pressure (mmHg)	14.2 (13.55)	11.7 (10.48)	0.208	0.033
IRP (mmHg)	2.75 (4.48)	3.58 (3.77)	0.028	0.774
UES resting pressure (mmHg)	50.9 (70.25)	59.6 (60.9)	0.03	0.759
UES residual pressure (mmHg)	7.5 (6.0)	7.1 (8.43)	0.122	0.211
DCI	1605.7 (743.75)	1140.1 (1056.6)	0.225	0.021
Normal contraction	10 (2)	8 (4.75)	0.201	0.039
Large defect contraction	0 (0)	0 (0)	0.037	0.702
Weak contraction	0 (1)	0 (1)	0.01	0.915
Ineffective contraction	0 (0)	0 (2)	0.208	0.03

Data are presented as median (interquartile range, IQR). Data are analyzed by Mann–Whitney *U*-test. Effect sizes: r. Higher LES resting pressure and DCI, more normal contraction and less ineffective contraction were observed in patients with SSD than those without SSD. LES: lower esophageal sphincter; IRP: integrated relaxation pressure; UES: upper esophageal sphincter; DCI: distal contractile integral.

### Risk factors for comorbid SSD in patients with GERD

**Figure 1:**
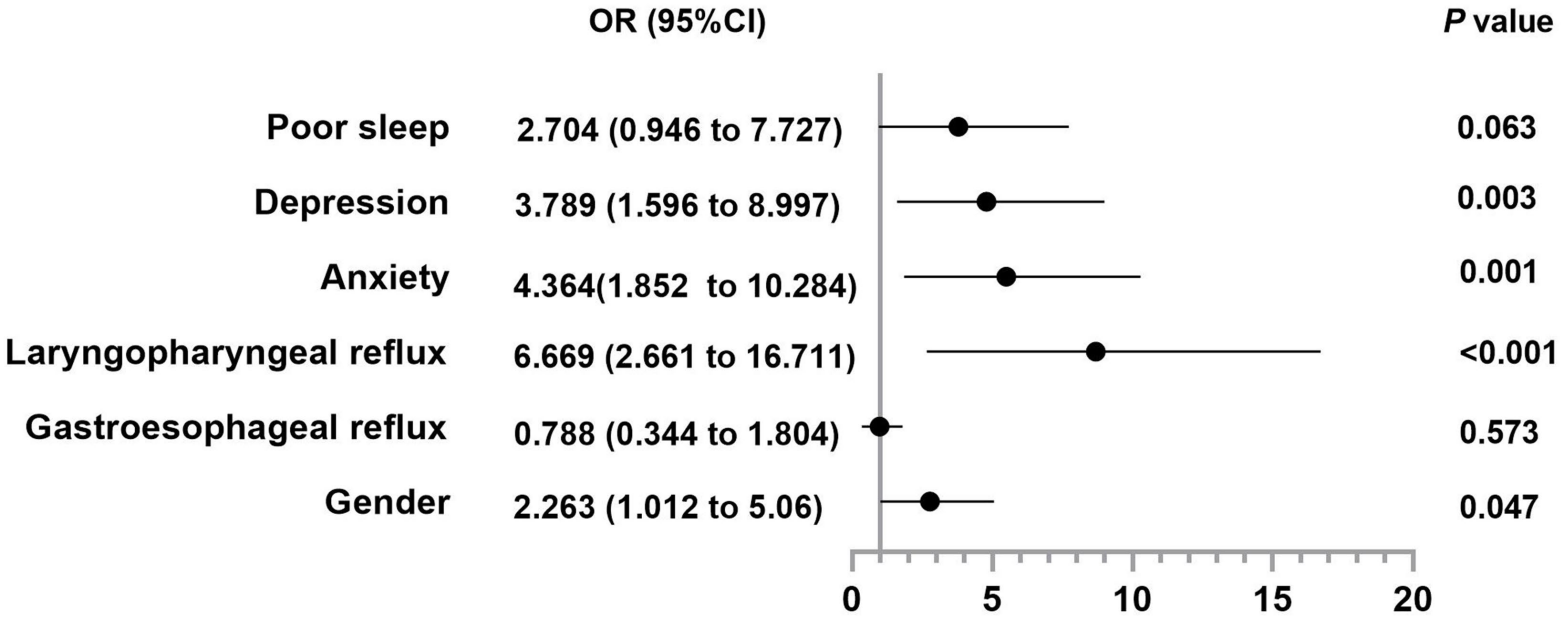
Forest plot suggesting the risk factors for SSD in patients with GERD. Data are analyzed by binary logistic regression.

Anxiety [GAD-7: 13(7) vs. 4.5(7), *p* < 0.001], depression [PHQ-9: 14(11) vs. 4(5), *p* < 0.001] and sleep disturbance [PSQI: 10(6) vs. 6(5), *p* < 0.001] were more prominent in SSD patients (Table 1). SSD severity (PHQ-15) positively correlated with gastroesophageal reflux (GerdQ with r = 0.236, *p* = 0.001) and laryngopharyngeal reflux symptoms (RSI with r = 0.508, *p* < 0.001), sleep disturbance (PSQI with r = 0.462, *p* < 0.001), anxiety (GAD-7 with r = 0.637, *p* < 0.001) and depression (PHQ-9 with r = 0.666, *p* < 0.001) symptoms. Female (OR = 2.263, 95% CI 1.012–5.06), laryngopharyngeal reflux (OR = 6.669, 95% CI 2.661–16.711), anxiety (OR = 4.364, 95% CI 1.852–10.284), and depression (OR = 3.789, 95% CI 1.596–8.997) emerged as independent risk factors for comorbid SSD (Figure 1). A multivariate regression model also identified them as the significant predictors of higher PHQ-15 scores (all *p* < 0.05, adjusted R^2^ = 0.581, VIF < 5).

### Impact of SSD on the reduced QOL in patients with GERD

**Figure 2:**
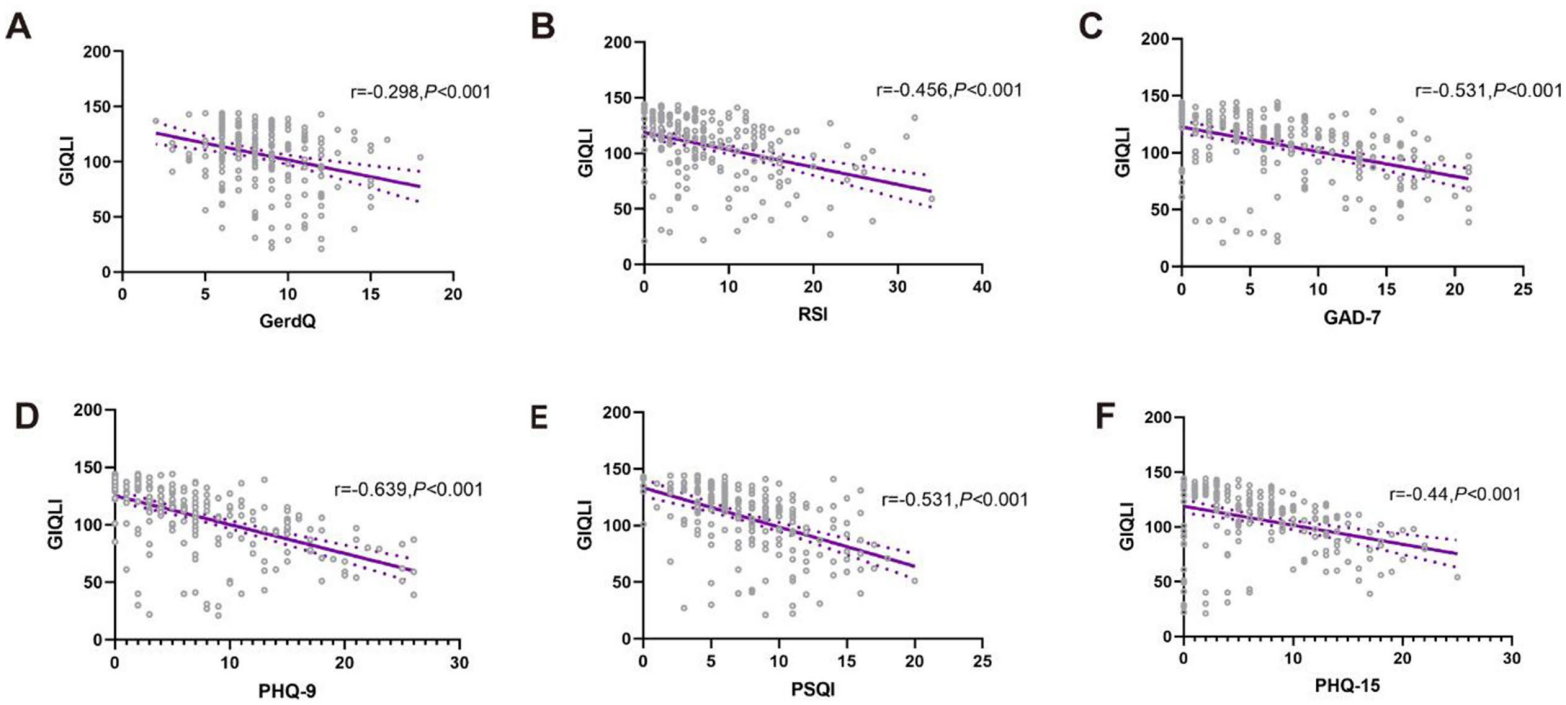
The correlations between QOL and reflux symptoms **(A,B)**, mental distress **(C,D)**, general sleep quality **(E)**, as well as somatic symptoms **(F)**. Data are analyzed by Spearman correlation analysis. GLIQI, Gastrointestinal Quality of Life Index; GerdQ, gastroesophageal reflux disease questionnaire; RSI, reflux symptom index; GAD-7, Generalized Anxiety Disorder 7-item; PHQ-9, 9-item Patient Health Questionnaire; PSQI, Pittsburgh Sleep Quality Index; PHQ-15, the Patient Health Questionnaire-15.

**Figure 3:**
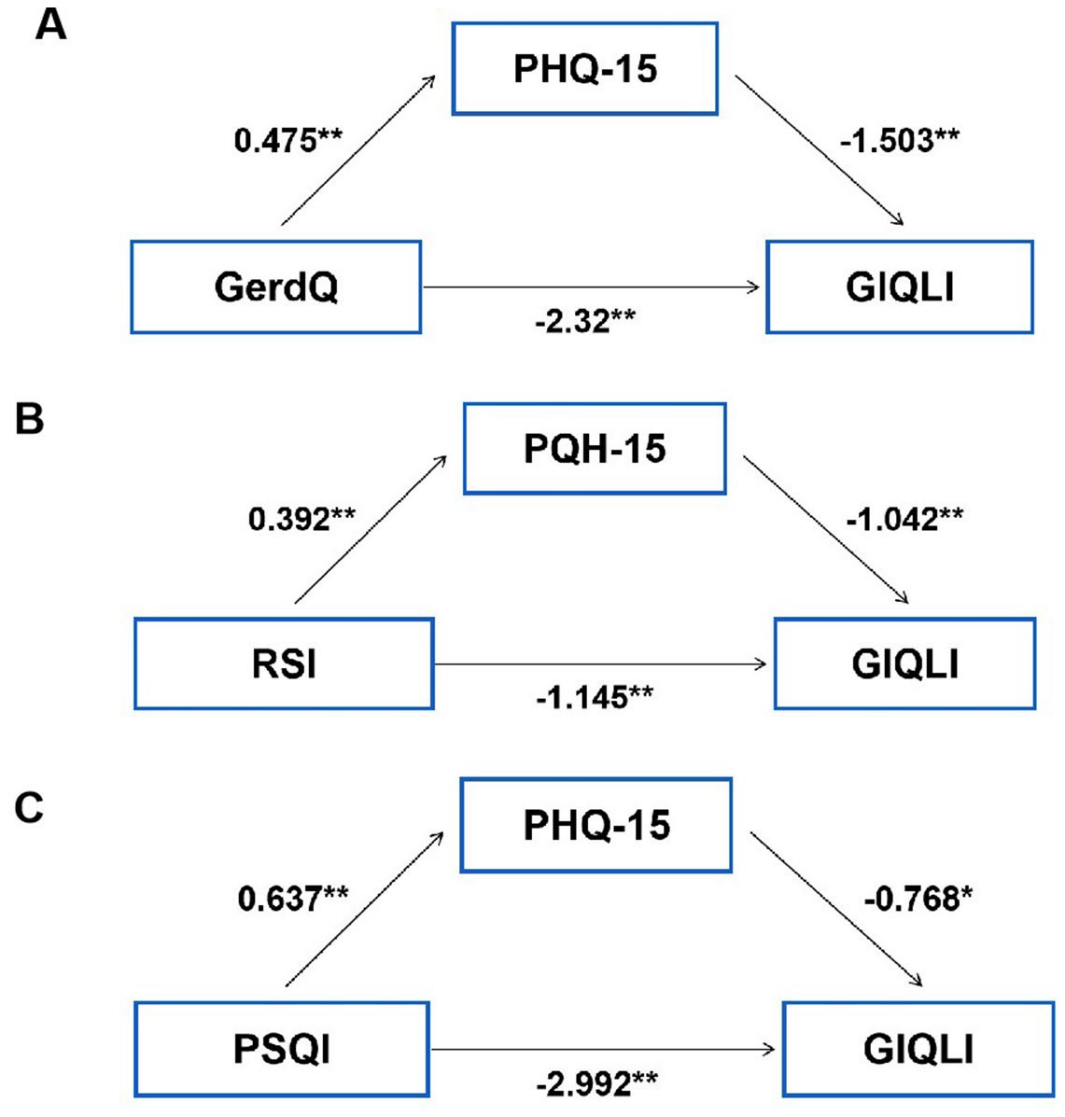
The mediating effects of SSD (PHQ-15) on QOL (GIQLI) impaired by gastroesophageal reflux (GerdQ) **(A)**, laryngopharyngeal reflux symptoms (RSI) **(B)**, and sleep disturbance (PSQI) **(C)**. Regression coefficients are displayed along the paths (**p < 0.01, *p < 0.05). GLIQI, Gastrointestinal Quality of Life Index; GerdQ, Gastroesophageal Reflux Disease Questionnaire; PHQ-15, The Patient Health Questionnaire-15; RSI, Reflux Symptom Index; PSQI, Pittsburgh Sleep Quality Index.

As shown in Table 1, QOL was significantly lower in the SSD group [GIQLI: 108(29) vs. 130(42), *p* < 0.001]. Reflux symptoms (GerdQ and RSI), anxiety (GAD-7), depression (PHQ-9), somatic symptom burden (PHQ-15), and sleep disturbances (PSQI) were all inversely correlated with QOL (GIQLI) (all *p* < 0.05; Figure 2). Figure 3 displayed indirect effects of reflux symptoms and sleep disturbance on reduced QOL through SSD. Specifically, the effect sizes were −0.713 for GerdQ, −0.409 for RSI, and −0.489 for PSQI (all *p* < 0.001). SSD accounted for 23.52, 26.30, and 14.05% of the total effects of gastroesophageal reflux (GerdQ), laryngopharyngeal reflux (RSI), and poor sleep quality (PSQI) on QOL impairment, respectively, indicating a partial mediation. In contrast, no such significant mediation effect of SSD was found for anxiety or depression.

### Clinical utility of SSD in screening psychological and sleep distresses

**Figure 4:**
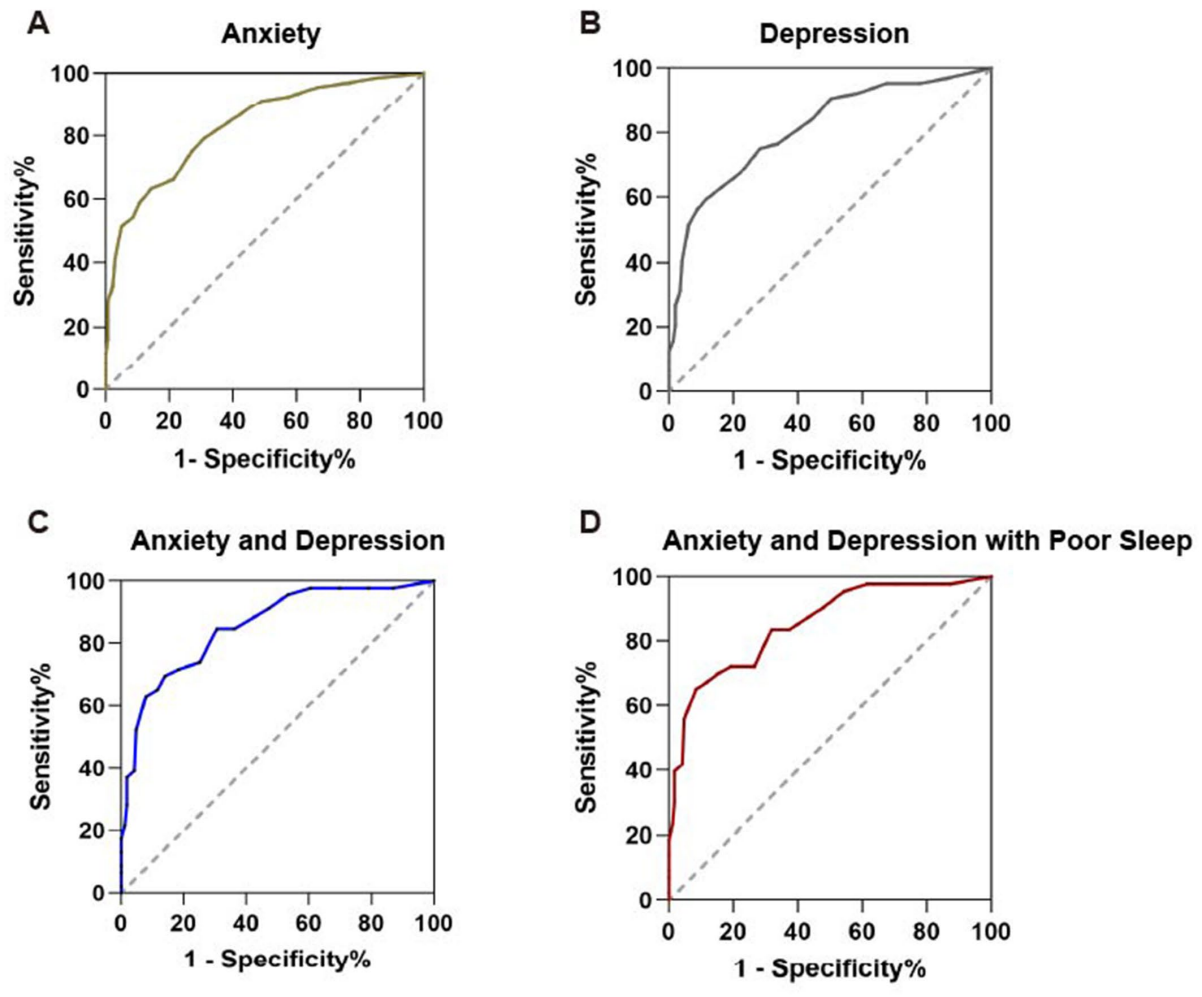
The diagnostic values of PHQ-15 for anxiety **(A)**, depression **(B)**, anxiety and depression **(C)**, anxiety and depression with sleep disorder **(D)**. Receiver operating characteristic (ROC) curves were used to assess the predictive value of PHQ-15.

In this population, 68.25% (43/63) and 63.49% (40/63) of SSD patients met the threshold for moderate anxiety (GAD-7 ≥ 10) and depression (PHQ-9 ≥ 10), respectively. ROC analysis demonstrated that the PHQ-15 has good predictive validity for detecting anxiety (AUC = 0.832; cutoff = 9.5, sensitivity 63.2%, specificity 85.8%) and depression (AUC = 0.812; cutoff = 10.5, sensitivity 59.4%, specificity 88.3%). For combined anxiety and depression, the AUC increased to 0.855 (cutoff = 10.5, sensitivity 69.6%, specificity 85.9%) (Figure 4). Sleep disturbance, common in individuals with psychological comorbidities, was strongly associated with SSD in this study. The PHQ-15 effectively identified patients with comorbid psychological distress (anxiety and depression) and poor sleep (PSQI > 5), yielding an AUC of 0.851 (cutoff = 12.5, sensitivity 65.1%, specificity 91.6%) (Figure 4).

## Discussion

Our study reveals three central findings for clinical practice. First, nearly one-third of patients with GERD meet the criteria for SSD, aligning with that 33.8% SSD patients were found in the outpatients from general hospital care (15). Reflux symptoms and sleep disturbances contribute to poor QOL, which is partly mediated by SSD. Second, despite reporting significantly higher symptom severity, patients with SSD exhibit milder objective reflux burden and nearly normal esophageal motility. This dissociation suggests that symptom perception in GERD is not solely determined by reflux evidence but is probably shaped by central sensitization and psychophysiological processes. Third, the PHQ-15 emerges as the practical instrument for assessing psychosomatic burden in patients with GERD.

SSD represents a diagnostic classification within mental disorders. Nevertheless, patients with this condition present a diverse range of non - specific symptoms, and are prone to being misdiagnosed as having organic diseases. The high prevalence of SSD, along with its robust correlation with poor QOL and psychological distress, underscores a necessity for screening SSD and implementing multidisciplinary management in clinical practice.

Psychosocial impairment is well-accepted with studies demonstrating remarkable QOL impairment and mental distress in patients with GERD (25). The marked discrepancy in our study between subjective symptom reports and objective reflux evidence in SSD patients suggests that mechanisms beyond acid exposure, such as visceral hypersensitivity and altered central nervous system processing of peripheral signals, play a critical role in symptom amplification. Brain–gut axis as a key modulator in gastrointestinal diseases, particularly among patients with comorbid anxiety and depression (26). A continuous bidirectional hormonal interaction between the central nervous system and the gastrointestinal tract suggests a direct relationship between GI stressors and heightened emotional and behavioral responses (27). Mental issues could lower perceptual thresholds and enhance reactivity to esophageal stimuli (28), explaining why patients with SSD report severe symptoms even without severe reflux burden. Comorbid SSD and other psychological problems may stem from a heightened response to reflux (2). The process through which esophageal events are perceived is a multifaceted process, dependent on both peripheral and central neurologic mechanisms. Although the precise details remain elusive, psychological stressors, cognitive-affective factors, and maladaptive coping behaviors are recognized as significant modulators of the overall symptom experience (29).

Notably, laryngopharyngeal reflux symptoms emerged as an independent risk factor for SSD, suggesting that extra-esophageal symptoms may be especially linked with somatization. And laryngopharyngeal reflux symptoms are reported to correlate with hypervigilance in a study (30). Therefore, a dual - pronged approach is needed: assessing central sensitization and psychological contributors while managing reflux with conventional treatments. Besides, depression and anxiety are independent risk factors for SSD. Psychological disturbances may aggravate GERD symptoms through shared pathophysiological mechanisms (31). Patients with depression suggest decreased sensory threshold and increased response to esophageal stimulation (12, 13). And poor response to proton pump inhibitor therapy is more common among patients with underlying anxiety or depression (32). Therefore, addressing anxiety and depression is essential in the management of GERD, as the presence of mental distress can complicate treatment and reduce therapeutic efficacy (27). We also found that sleep disturbance is significantly prominent in patients with SSD. Clinical evidence indicates that sleep disturbance induces esophageal hyperalgesia to acid exposure, thereby amplifying GERD symptoms (33). In the present study, SSD was strongly associated with impaired sleep quality in addition to reflux symptoms, presenting additional challenges for both patients and physicians.

Additionally, our results suggest a higher prevalence of SSD among females than males. This observation is consistent with general population studies indicating that adolescent girls are more likely than boys to report persistent somatic symptoms (34). Females with SSD exhibit reduced vagal activity in response to somatic stimuli compared to those without SSD, a phenomenon not observed in men (35). Moreover, female sex has been identified as an independent risk factor for SSD in older adults (36). In general, the differences between genders may be related to the biological basis, coping styles, and socio-cultural factors (37, 38). The gender difference warrants further investigation to clarify underlying mechanisms.

QOL in patients with GERD exhibits an inverse correlation with reflux severity, somatic symptoms, anxiety, depression and sleep disturbance. A complex interplay exists among these factors (39). Patients are often trapped in a vicious cycle which leads to substantial deterioration of QOL. Mediation model examines whether the relationship between an independent variable and an outcome is explained by an intervening variable, known as a mediator (40). Our analysis identifies SSD as a critical mediator that exacerbates the detrimental effects of reflux symptoms and sleep disturbance on QOL. Consequently, effectively addressing SSD is essential for achieving comprehensive improvements in patients’ QOL.

Based on the findings of our study, the high prevalence of SSD (30.14%) and its role as a key mediator necessitate a paradigm shift in GERD management. An integrated, multidisciplinary care model is warranted. Routinely screening for SSD, particularly in high-risk subgroups such as females and patients with prominent laryngopharyngeal reflux symptoms, is crucial. Effective management must extend beyond acid suppression to address the central sensitization and maladaptive cognitive-behavioral patterns characteristic of SSD. Notably, PHQ-15 could be a convenient instrument, which is used to efficiently recognize patients with significant somatic symptom burden (16). Its brevity and reliability make it valuable in non-psychiatric medical settings. We propose a stepped-care model wherein GERD patients with high PHQ-15 scores (≥12.5) are referred for psychological evaluation and integrated treatments. This multidisciplinary collaboration between gastroenterologists and psychologists is essential to disrupt the vicious cycle of GERD symptoms, psychological distress, and poor QOL, ultimately improving patient-centered outcomes.

Our study adopted the well-established diagnostic criteria for GERD and SSD and revealed the clinical value of identifying SSD timely. However, several limitations should be acknowledged. The single-center design may limit generalizability, and the cross-sectional nature of our data precludes causal inference. Future prospective and multicenter studies should validate the utility of PHQ-15 in broader populations and investigate the outcomes of integrated treatment models. Besides, incorporating functional neuroimaging techniques such as functional MRI could provide detailed insights into mechanisms of central sensitization. Lastly, other confounding factors impacting GERD symptoms and related QOL are not thoroughly examined.

## Conclusion

In summary, SSD is a key driver of the disproportionate symptom severity in GERD. It functions as a prominent mediator exacerbating the negative effects of reflux symptoms and sleep disturbances on QOL. The PHQ-15 serves as an efficient instrument for identifying high-risk patients, and integrating SSD screening into GERD management is essential for holistic patient care.

## Data Availability

The original contributions presented in the study are included in the article/supplementary material, further inquiries can be directed to the corresponding author.
